# Psychological readiness to return to sports practice and risk of recurrence: Case studies

**DOI:** 10.3389/fpsyg.2022.905816

**Published:** 2022-09-23

**Authors:** Veronica Gomez-Espejo, Aurelio Olmedilla, Lucia Abenza-Cano, Alejandro Garcia-Mas, Enrique Ortega

**Affiliations:** ^1^Department of Psychology, University of Murcia, Murcia, Spain; ^2^Department of Personality, Evaluation and Psychological Treatment, University of Murcia, Murcia, Spain; ^3^Faculty of Sport, Catholic University of Murcia, Murcia, Spain; ^4^Grupo de Investigación en Ciencias de la Actividad Fisica (GICAFE) (Research Group of Sports Sciences), University of the Balearic Islands, Palma de Mallorca, Spain; ^5^Department of Physical Activity and Sport, Campus of Excellence Mare Nostrum, University of Murcia, Murcia, Spain

**Keywords:** psychological readiness, iceberg profile, RTP, sport injury, recurrence

## Abstract

Returning to sport after the sports injury is a difficult decision because it’s multicausal and the fact that a rash decision can result in numerous negative consequences. Given the importance of psychological variables for the correct rehabilitation of the injured athlete and his or her optimal return to sports practice, there seems to be little information on this subject. In this sense, the objective is to determine the relationship between the subjective psychological disposition of the athlete in the process of Return to Play (RTP) with the type of mood profile and his mental health. This is based on the fact that each athlete evaluates his or her recovery differently and has different levels of anxiety, depression, and stress. For this purpose, four athletes participated in the study. Two males and two females from the sports of indoor soccer and soccer, who had just returned to sports after a moderate or severe injury. The average age was 24.25 years. Various measurements were taken after practices and after matches, to assess mood, psychological readiness, anxiety, stress, and depression. The results confirm Morgan’s iceberg profile and the influence that subjective psychological perceptions and assessed emotional states have on athletes’ incorporation into their sports practice with a guarantee of success.

## Introduction

Sports injuries have a negative emotional impact on the health and performance of the affected athlete and result in high health and sports economic cost (Palmi, 2014; Emery and Pasanen, 2019; O’Brien et al., 2019; Kvist and Silbernagel, 2022). After a sports injury, the athlete undergoes a physical rehabilitation process (Baez et al., 2020; Goddard et al., 2020; Kvist and Silbernagel, 2022). Thanks to technological and medical advances, 90% of athletes undergo rehabilitation regain normal function of the injured area; but only 63% of them return to pre-injury levels and 44% return to competition (Ardern et al., 2011). These results suggest that factors other than the physical play a role in a successful return to sport. In this sense, after the rehabilitation process is completed, the decision is made whether the injured athlete can return to sport (Conti et al., 2019; Green et al., 2020).

For years, researchers have pointed out the importance of psychological factors in sports injury susceptibility (García-Mas et al., 2014; Ganz, 2018; Slimani et al., 2018) and their recovery (Casáis, 2008; Arvinen-Barrow and Clement, 2016; Salim et al., 2016; Roy-Davis et al., 2017; Olmedilla et al., 2018a; Gennarelli et al., 2020). Several theoretical models suggest that personality, coping resources and emotional state influence sports injuries (Andersen and Williams, 1988; Olmedilla and García-Mas, 2009; Johnson and Ivarsson, 2011; Assa et al., 2018; Meierbachtol et al., 2018; Arroyo del Bosque et al., 2020).

There are numerous references that establish relationships between sports injury and the athlete’s psychological predisposition to resume sports practice (Chomiak et al., 2000; Forsdyke et al., 2017; Zarzycki et al., 2018; Kunnen et al., 2019; Cheney et al., 2020). For this reason, based on the emotional pattern “U” experienced during the injury process, which maintains the occurrence of negative responses both at the beginning and at the end of the process (Morrey et al., 1999; Ardern et al., 2017), numerous authors have emphasized the importance of a good psychological predisposition before Return to Play (RTP) (Christino et al., 2016; Burland et al., 2018; Caron et al., 2018; Kitaguchi et al., 2019; Ashton et al., 2020). In this sense, lack of psychological preparation has been identified as a factor preventing proper RTP (Risberg et al., 2007; Ardern et al., 2014; Nwachukwu et al., 2019) and may persist even when physical disabilities have been resolved (Ross, 2010; Lentz et al., 2015; Gennarelli et al., 2020).

The Sports Medicine Council (2002) defines Return to Play as the point at which the injured athlete makes the decision to safely return to training and competition (Herring, 2002). Some authors (Hammond et al., 2013; Haitz et al., 2014; Burland et al., 2018; Rollo et al., 2021), caution that the RTP can sometimes be “unreal” as external variables such as environmental pressures coach request, fan demand or the injured athlete’s urge to not lose status can lead to a hasty decision. For this reason, any sports injury should be considered from the global framework of the athlete.

The emotional states experienced by the injured athlete during this period of recovery from the sports injury will help determine RTP in one way or another. Specifically, some studies suggest that stress can lead to increased risk of injury and influence their recovery (Nippert and Smith, 2008; De la Vega et al., 2013; Heidari et al., 2016; Olmedilla and García-Mas et al., 2017; Olmedilla et al., 2018b; Leguizamo et al., 2021). Similarly, depression and “low” mood have received particular attention (Purcell et al., 2019). However, knowledge about the relationship between psychological factors and sports injuries is still limited (Olmedilla et al., 2018b; Gouttebarge et al., 2019; Rice et al., 2019). Therefore, it’s likely that injured athletes with positive emotional responses achieve better rehabilitation, which would positively correlate with a correct RTP (Sabato et al., 2016; Gomez-Espejo et al., 2018; Nwachukwu et al., 2019; Rollo et al., 2021). Therefore, athletes who are ready for the RTP are more likely to have better emotional responses. Therefore, any apprehension the athlete feels while preparing for the RTP may indicate that rehabilitation is incomplete (Cheney et al., 2020). Conversely, athletes who are not psychologically prepared for the RTP are less likely to return to sport. And those who do return to sport may be at increased risk for recurrence, poor athletic performance and lower-quality of the sport experience (Ardern et al., 2013, 2014; Czuppon et al., 2014; Podlog et al., 2015; Baugh et al., 2017; Brewer, 2017; Green et al., 2020). Therefore, the fact of suffering a sports injury is particularly relevant as it not only represents a physical problem, but also implies a change in the psychological disposition of the athlete. For this reason, Morgan developed his own Mental Health Model (Morgan, 1980), according to which successful athletes have more positive and less negative mental health characteristics than less successful athletes and the general population. The iceberg profile would essentially be the profile of a mentally healthy person (Andrade et al., 2016, 2019; Terry and Parsons-Smith, 2021).

Thus, when returning to sport, athletes express concerns about the prospect of recurrence (Ardern et al., 2013; Flanigan et al., 2013; Czuppon et al., 2014; Brewer, 2017; Meierbachtol et al., 2018; McPherson et al., 2019), have decreased performance or execution ability (Podlog et al., 2013), have deficits in intrinsic motivation to return to their sport (Brewer, 2010, 2017; Ardern et al., 2013; Czuppon et al., 2014; Hamrin-Senorski et al., 2017; Slagers et al., 2017), and they appear physically unable to return to sport (Ardern et al., 2013; Podlog et al., 2013; Czuppon et al., 2014; Brewer, 2017; Hamrin-Senorski et al., 2017; Slagers et al., 2017).

In this sense, RTP and the potential risk of recurrence are often as emotional events as the injury itself and are identified as potential limiting factors for rehabilitation and successful RTP (Creighton et al., 2010; Milewski et al., 2016; Cheney et al., 2020). Although psychological interventions improve sports injury function, it is unknown how psychological preparation influences the risk of a second injury (Lentz et al., 2018). Several studies (Paterno et al., 2012; Webster et al., 2014; Wiggins et al., 2016; Zhang et al., 2022) have shown that many athletes who return to their previous activity level sustain a second injury, demonstrating the importance of psychological health (McPherson et al., 2019). Therefore, it’s necessary to develop specific strategies to facilitate decision making about the ideal time for an injured player to the return to sport (Gómez-Piqueras et al., 2013; O’Brien et al., 2017; Tjong et al., 2017; Webster et al., 2017; Gómez-Espejo et al., 2018; McCrory et al., 2018; McPherson et al., 2019), understanding this as an ongoing decision-making process that needs to be dynamic and personalized (Ekstrand and Gillquist, 1983; Pruna, 2016; Cheney et al., 2020). Although there is currently consensus on the need to examine the physical and psychological factors surrounding RTP (Feller and Webster, 2013; Ivarsson et al., 2013; Takeshita et al., 2016; Forsdyke et al., 2017; Webster et al., 2017, 2018; Lai et al., 2018; Welling et al., 2018; Werner et al., 2018; Baez et al., 2019; Kaplan and Witvrouw, 2019), existing criteria do not comprehensively consider psychological preparation for competition.

For this reason, it is necessary to include strategies for the correct follow-up of the injured that allow to objectify the decisions of the professionals (Gómez-Piqueras et al., 2014, 2020), since, according to Glazer (2009) and Webster et al. (2008), it has been noted that today there are not enough instruments that evaluate in a specific way the psychological predisposition of the injured in the moment before the reappearance, and that include specific questions about this phase and the specifics of the injury. In this sense, Gómez-Piqueras et al. (2014) developed an instrument that measures the perception of the injured athlete in relation to his return to training after an injury, which proved to be effective for this purpose.

The aim of this study is to determine the relationship between pre-RTP subjective psychological disposition and mental health indicators in four cases of injured soccer and futsal players before a pandemic and lockdown situation.

## Materials and methods

### Participants

The following inclusion criteria were established for participation in the study: Athletes had to have been discharged by a physician less than one week ago, have sustained a severe or moderate sports injury, not have a chronic physical or mental illness.

Considering all the inclusion criteria and the athlete population that meets them, the work was carried out with 4 soccer players (2 soccers and 2 futsal players) from different sports categories belonging to sports clubs. The average age of the athletes was 24.25 years, with an age range between 18 and 28 years. The average number of years they practiced their sport in the highest category was 2.5 years, while the average number of years they practiced the sport continuously was 11 years.

In terms to to the type of sports injury sustained, the inclusion criteria for the study were: that it was a recently rehabilitated sports injury (return to sport two days prior to the initial evaluation), that it was new (no recurrences or relapses) and that it was medically diagnosed as moderate or severe. That is, they were a sports injury with an estimated recovery time of at least 15 days of treatment. The characteristics of the remaining participating subjects to be included in the study are listed below:

*Subject 1*: 18-year-old male, professional soccer player. He plays as a goalkeeper. He trains 3 days a week, averaging 5 h a week. He suffered a knee sprain that forced him to miss 17 training sessions and 6 games in a row.

*Subject 2*: 26-year-old female, professional soccer player. She plays as a striker. She trains 3 days per week with an average of 6 h per week. She suffered a dislocated shoulder that forced her to miss 15 training sessions and 5 games in a row.

*Subject 3*: 25-year-old male, futsal player. He plays in the position of goalkeeper. He trains 3 days per week, averaging 6 h per week. He suffered from shoulder tendinitis, which forced him to miss 12 training sessions and 3 games in a row.

*Subject 4*: 28-year-old female, futsal player. She plays as a wing player. He trains 4 days per week, averaging 8 h per week. He suffered a torn meniscus, which forced him to miss 22 training sessions and 6 games in a row.

### Instruments and materials

The psychological assessment instruments used for the study were:

*Personal and sports variables questionnaire. Ad hoc* questionnaire to collect the athlete’s socio-demographic data (see Annex I).

*History of sports injuries*. *Ad hoc* created questionnaire, based on an injury protocol (Olmedilla and García-Mas et al., 2017). It captures the number of sports injuries sustained in the last two seasons and specific data about them (see Annex II).

*Profile of Mood States* (POMS, McNair et al., 1971). In its Spanish version adapted and validated by Fuentes et al. (1995). It is a self-report questionnaire for measuring mood. The short version was used, with 29 items answered on a Likert-type scale with 5 response options. It includes 5 dimensions: Tension (α = 0.83); Depression (α = 0.78); Anger (α = 0.85); Vigor (α = 0.83); Fatigue (α = 0.82).

*Depression, Anxiety and Stress Scale-21items* (DASS-21, Lovibond and Lovibond, 1995). In its version adapted and validated in Spanish (Fonseca-Pedrero et al., 2010). Has been used to measure general symptoms of depression, anxiety and stress. This scale has three subscales: depression, anxiety and stress, each consisting of 7 items, for a total of 21. In a Likert-type response scale, each item has four response options. It has a Cronbach’s Alpha of 0.81.

*Psychological Readiness of Injured Athlete to return to sport* (PRIA, Gómez-Piqueras et al., 2014). The assessment instrument consists of 10 questions/items that include statements about self- confidence, individual status, uncertainty and fear of relapse. Scores range from 1 to 5, with higher scores corresponding to better psychological disposition.

### Procedure

Prior to the psychological assessment, the rehabilitation staff Football Federation of the Region of Murcia (FFRM) was contacted directly, thanks to a collaboration agreement in place at the time between the University of Murcia (UMU) (an organization in which the psychologist in charge of the study worked) and the FFRM. The rehabilitators served as a link between the psychologist and the athletes, providing the contact details of those athletes who met the inclusion criteria. The purpose of the study and the procedure were explained to the rehabilitators and later to the participating injured athletes (via telephone). In addition, all participants were informed of the purpose of the study and the confidentiality of both their responses and previously collected data. Informed consent and the privacy document were obtained from all participants. The entire evaluation process and subsequent contact was conducted online.

The study was approved under research ethics by the Ethics Committee of the University of Murcia (Spain), with reference number CEI-2623-2019. The moment the athletes were medically discharged from the FFRM, the psychological evaluation by the psychologist in charge of the project began. The evaluation was done online and consisted of three different moments related to the return to play (RTP), so the psychological evaluation process was as follows:

Initial assessment. It’s conducted immediately after medical discharge. At this time, an assessment battery consisting of personal and sports variables, PRIA questionnaire and POMS questionnaire was used. For this purpose, an assessment battery was sent online via email, which could be completed directly by clicking on the link.

Monitoring of training. Completed once a week after practices. Recording the date and time of training, as well as the POMS.

Tracking of games. Completed after each match in which the athlete was used. Recorded the POMS, the DASS-21 and the PRIA.

It should be noted that the evaluation process was interrupted earlier than planned when competitions and training were interrupted due to the state of alert and lockdown declared by the Spanish Government because of the Covid-19 pandemic.

### Data analysis

Descriptive statistics were used for data analysis, employing counts, sums, percentages and measurements. The results of the POMS questionnaire were converted to a scale of 0 to 100 points, with 50 being the mean. Participants’ pre-competition mood profile were analyzed and described. The graphs of the pre-competition and pre-training mood profiles were created. Likewise, the graphs were made with the results of the PRIA questionnaire. The statistical program SPSS 22.0 was used.

## Results

### Subject 1 results

**FIGURE 1:**
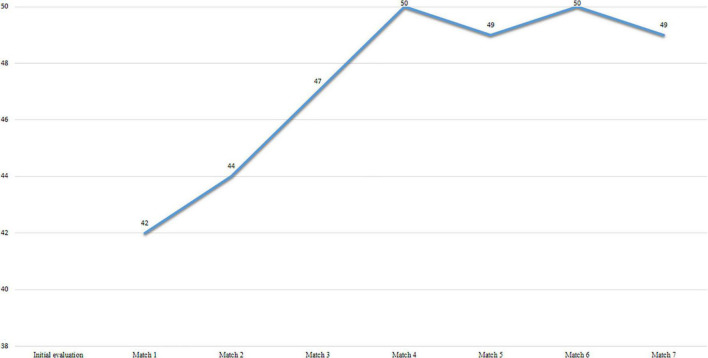
Subject 1 PRIA scores.

Figure 1 shows the scores obtained in the Questionnaire of psychological predisposition of the injured athlete (PRIA) during the evaluation. Scores above 40 indicate that the athlete’s psychological disposition is sufficient to return to play with some degree of confidence. The lowest value of 42 points means that the athlete is psychologically ready to return to play. Moreover, it can be observed that the psychological predisposition increases progressively until the third game and then stabilizes.

Table 1 shows the values that Subject 1 obtained on the POMS factors in the seven shots evaluated after the matches.

**TABLE 1:** Values in the POMS factors of Subject 1 after the matches.

Time of evaluation	Tension	Anger	Vigor	Fatigue	Depression
Match 1	4.2	21.8	100	10	0
Match 2	4.2	12.5	60	5	5
Match 3	20.8	0	80	5	0
Match 4	12.5	12.5	95	5	0
Match 5	0	12.5	100	0	0
Match 6	16.7	12.5	100	0	0
Match 7	0	12.5	80	0	0

**FIGURE 2:**
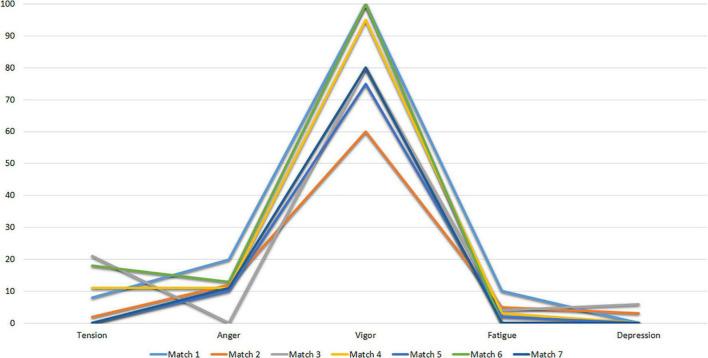
POMS profiles of subject I after matchs.

Figure 2 shows the mood profiles of Subject 1 at the evaluation time points after the competition (Match 1 to Match 7).

The results of these figures correspond to the iceberg profile described by Morgan (1980), which shows no change in mood states before the competition. In this sense, the profile obtained by Subject 1 is characterized by low scores in Tension, Anger, Fatigue and Depression and a level in the Vigor factor above the central value (50).

Table 2 shows the values obtained by Subject 1 in the POMS factors in the seven shots evaluated weekly after training.

**TABLE 2:** Values in the POMS factors of Subjetc 1 after training.

Time of evaluation	Tension	Anger	Vigor	Fatigue	Depression
Training week 1	4.2	15.6	90	10	0
Training week 2	4.2	12.5	60	5	5
Training week 3	25	0	90	5	0
Training week 4	12.5	12.5	95	5	0
Training week 5	4.2	15.6	100	0	0
Training week 6	12.5	9.4	100	0	0
Semana entreno 7	0	12.5	80	0	0

**FIGURE 3:**
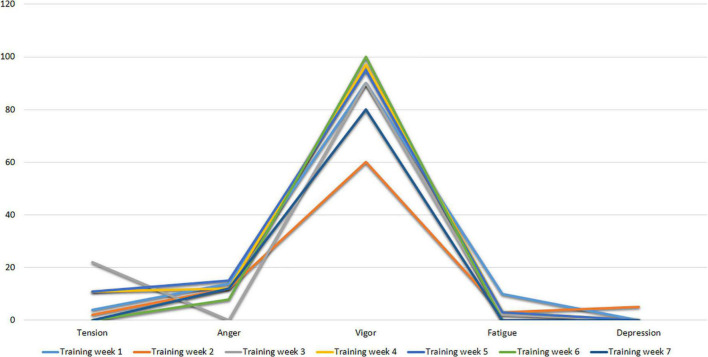
POMS profiles of subject I after training weeks.

Figure 3 shows the profiles of Subject 1 in the first three assessment time points after training (Training Week 1 to Training Week 7). As can be seen, Subject 1 shows an ideal pre-competition mood profile, consistent with the iceberg profile, where the Vigor factor is higher than the other factors and above the central value (50 points).

**FIGURE 4:**
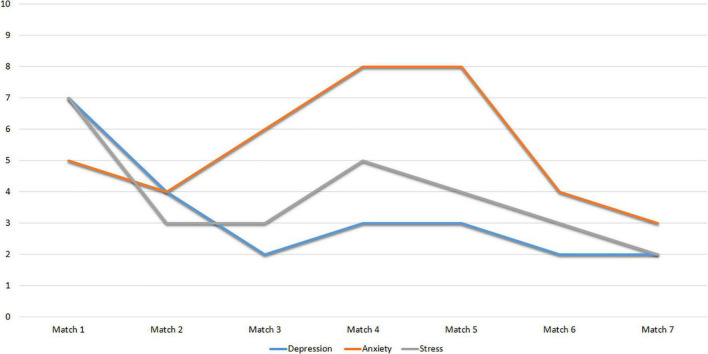
Scores of subject 1 to the subscales of the DASS-21.

Finally, Figure 4 shows the scores obtained by Subject 1 in the DASS-21 subscales. It shows indicators of adequate mental health, with punctual severe anxiety peaks.

### Subject 2 results

**FIGURE 5:**
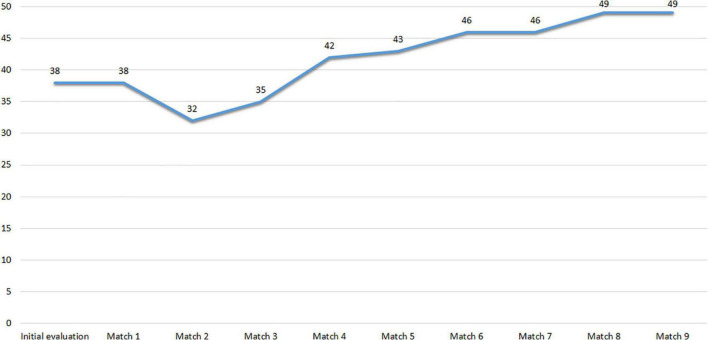
Subject 2 PRlA scores.

Figure 5 shows the scores obtained by Subject 2 on the PRIA throughout the assessment. The results show that until Match 3, the athlete’s predisposition to return to sport was not sufficient or other types of complementary testing should be considered. From Match 4, the scores show adequate psychological predisposition to return to sport with some guarantees.

Table 3 shows the values of the POMS factors of Subject 2 in the nine recordings evaluated after the matches.

**TABLE 3:** Values in the POMS factors of Subject 2 after the matches.

Time of evaluation	Tension	Anger	Vigor	Fatigue	Depression
Match 1	58.3	18.7	75	5	0
Match 2	25	12.5	75	0	0
Match 3	25	9.4	75	0	0
Match 4	25	15.6	75	0	0
Match 5	16.7	15.6	75	0	0
Match 6	4.2	9.4	85	0	0
Match 7	20.8	9.4	75	5	0
Match 8	4.2	12.5	70	0	0
Match 9	16.7	12.5	60	0	0

**FIGURE 6:**
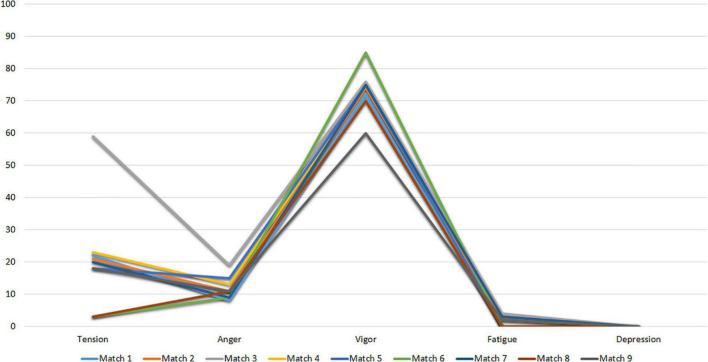
POMS profile of subject 2 after matchs.

Next, Figure 6 shows the mood profiles of Subject 2 after the competition (Match 1 to Match 9). The results of Figure 6 show that Subject 2 gradually adopts an iceberg mood profile throughout the evaluation period. In this sense, the profile is characterized by low scores on the Tension, Anger, Fatigue and Depression factors. Vigor shows values above 50 (mean) in all the evaluated recordings.

Table 4 shows the scores that Subject 2 obtained on the POMS factors in the nine shots evaluated weekly after training.

**TABLE 4:** Values in the POMS factors of Subjetc 2 after training.

Time of evaluation	Tension	Anger	Vigor	Fatigue	Depression
Training week 1	20.8	12.5	75	5	0
Training week 2	16.7	12.5	75	0	0
Training week 3	20.8	9.4	85	0	0
Training week 4	25	15.6	75	0	0
Training week 5	16.7	12.5	80	0	0
Training week 6	4.2	9.4	90	0	0
Training week 7	20.8	9.4	75	5	0
Training week 8	4.2	12.5	70	0	0

**FIGURE 7:**
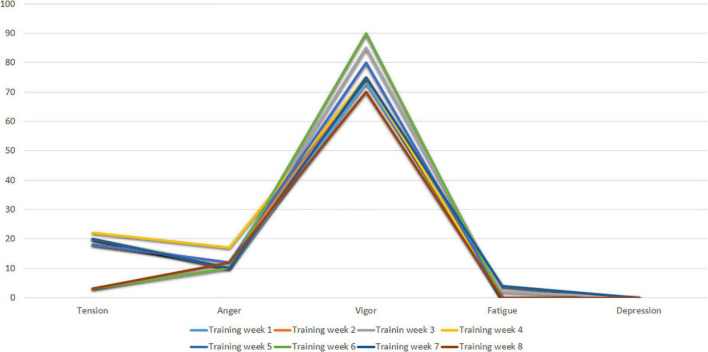
POMS profile of subject 2 after training weeks.

Figure 7 shows the profiles of Subject 2 after training (Training week 1 to Training week 8). The results of the following Figure show that Subject 2 has a good pre-competitive mood profile, which is consistent with the iceberg profile. It shows an adequate state of coping with the competition, where the Vigor factor is above the central value (50 points) and the other factors are perfectly leveled for a correct athletic performance.

**FIGURE 8:**
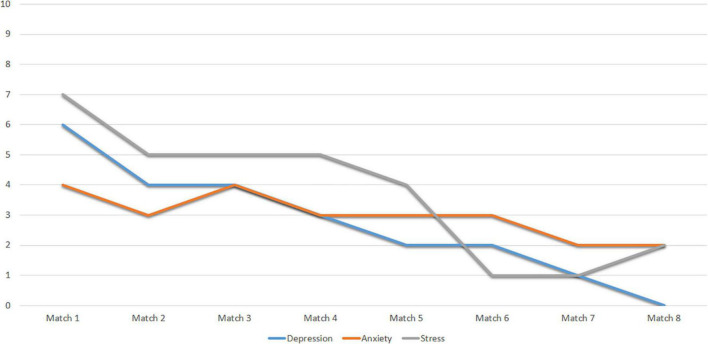
Scores of subject 2 to the subscales of the DASS-21.

Finally, Figure 8 shows the scores obtained by Subject 2 on the DASS-21 subscales. The score profile shows indicators of adequate mental health, with slight expressions in all factors.

### Subject 3 results

**FIGURE 9:**
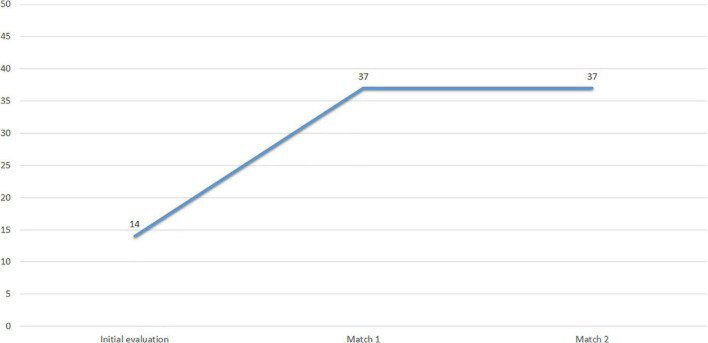
Subject 3 PRlA scores.

Figure 9 shows the scores obtained by Subject 3 at PRIA throughout the assessment. The results indicate that the psychological predisposition to return to sport was not sufficient in the first assessment, and although it has higher scores in Matches 1 and 2, the psychological predisposition of this athlete is uncertain and additional testing is needed to determine it.

Table 5 shows the values of the POMS factors of Subject 3 in the two recordings evaluated after the matches.

**TABLE 5:** Values in the POMS factors of Subject 3 after the matches.

Time of evaluation	Tension	Anger	Vigor	Fatigue	Depression
Match 1	50	46.8	40	55	55
Match 2	16.7	12.5	85	0	0
Match 3	12.5	9.4	80	0	0

Figure 9 shows the profiles of Subject 3 in the three evaluation time points after the competition (Match 1 to Match 3).

Figure 9 shows a pre-competitive mood profile suitable for coping with competition. It shows an adequate iceberg profile in Matches 2 and 3. However, the mood profile of Match 1 is very inconsistent and shows almost an inverted iceberg profile, with a Vigor value lower than that of the other variables.

Table 6 shows the values of the POMS factors of Subject 3 in the two weekly post-training recordings evaluated.

**TABLE 6:** Values in the POMS factors of Subjetc 3 after training.

Time of evaluation	Tension	Anger	Vigor	Fatigue	Depression
Training week 1	16.7	15.6	85	45	0
Training week 2	16.7	15.6	85	5	0

**FIGURE 10:**
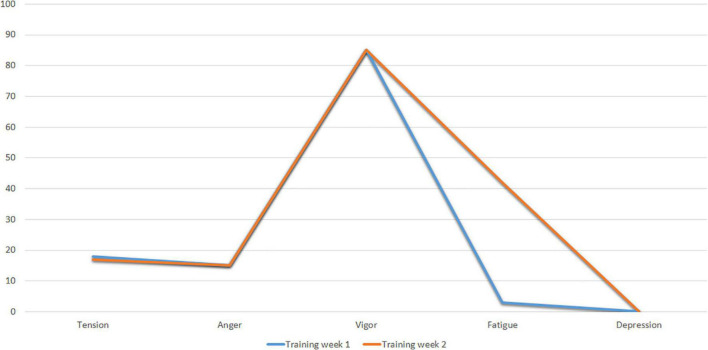
POMS profile of subject 3 after training weeks I and 2.

Figure 10 shows the profiles of Subject 3 at the two post-training evaluation times (Training Week 1 and Training Week 2). The results of Training Week 1 show a profile of pre-competitive mood suitable for competition, while Training Week 2 shows a Fatigue level higher than expected.

Finally, the scores obtained by Subject 3 on the DASS-21 subscales show a score of 2 points in Anxiety in Match 1 and 6 points in Match 2. In Depression, it shows a score of 5 in Match 1 and 2 points in Match 2. In stress, the athlete gets 5 points in Match 1 and 0 points in Match 2. The data show signs that this athlete’s mental health is not adequate, as the depression, anxiety, and stress scores increase as the evaluation progresses.

### Subject 4 results

**FIGURE 11:**
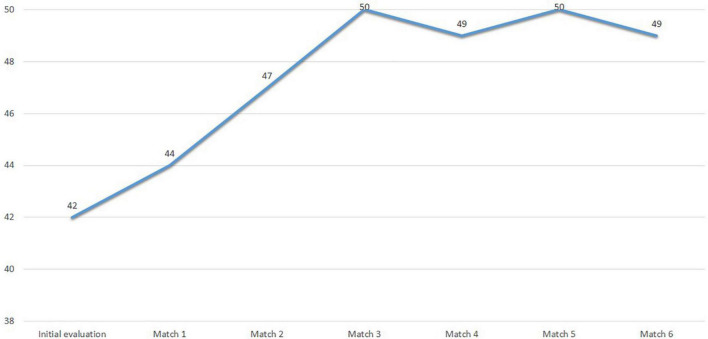
Subject 4 PRIA scores.

Figure 11 shows the scores obtained by Subject 4 at PRIA. Scores above 40 indicate that the athlete is ready to return to sports with confidence.

Table 7 shows the scores of the POMS factors of Subject 4 in the seven recordings evaluated after the matches.

**TABLE 7:** Values in the POMS factors of Subject 4 after the matches.

Time of evaluation	Tension	Anger	Vigor	Fatigue	Depression
Match 1	50	43.7	70	45	20
Match 2	8.3	21.8	90	0	0
Match 3	12.5	9.4	75	0	0
Match 4	12.5	12.5	90	0	0
Match 5	4.2	9.4	85	0	0
Match 6	4.2	12.5	60	0	0

**FIGURE 12:**
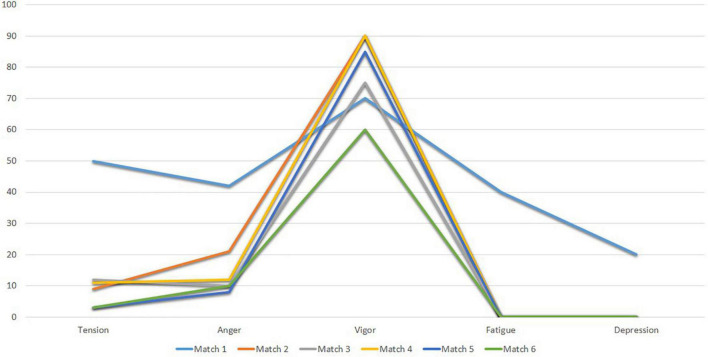
POMS profiles of subject 4 after matchs.

Next, Figure 12 shows the mood profiles of Subject 4 in Match 1 (Match 1 to Match 6). The pre-competitive mood profile from Matches 2 to 6 shows that it’s consistent with the iceberg profile. Here, the Vigor factor is above the mean (50 points). However, the mood profile for Match 1 deviates slightly from the iceberg profile.

Table 8 shows the values of the POMS factors of Subject 4 in the seven shots evaluated weekly after training.

**TABLE 8:** Values in the POMS factors of Subject 4 after training.

Time of evaluation	Tension	Anger	Vigor	Fatigue	Depression
Training week 1	4.2	18.7	70	45	5
Training week 2	8.3	21.8	90	0	0
Training week 3	16.6	9.4	75	0	0
Training week 4	12.1	12.5	90	0	0
Training week 5	4.2	9.4	85	0	0
Training week 6	4.2	9.4	65	0	0
Training week 7	4.2	6.2	85	0	0

**FIGURE 13:**
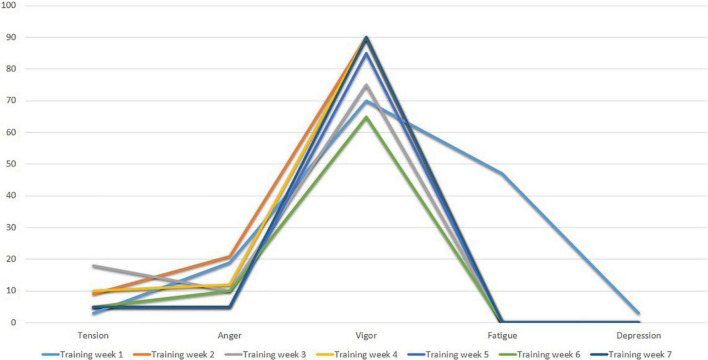
POMS profiles of subject 4 after training weeks.

Figure 13 shows the mood profiles of Subject 4 in Training Weeks (Training Week 1 to Training week 7). Subject 4 shows an adequate pre-competitive mood profile in which the Tension, Anger, Fatigue and Depression factors have low values, while the Vigor factor has high values. Characteristic scores of the iceberg profile indicating an appropriate state of mind for the competition.

**FIGURE 14:**
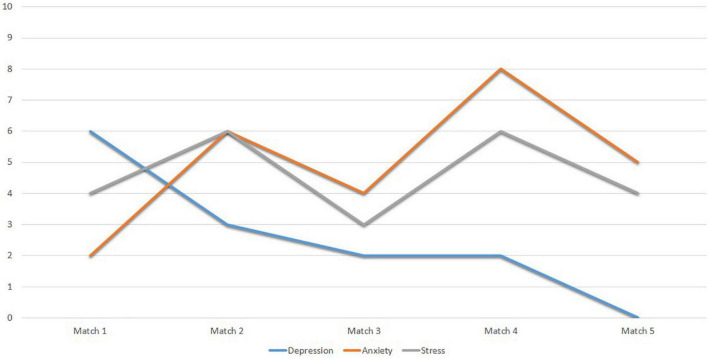
Scores of subject 4 to the subscales of the DASS-21.

Finally, Figure 14 shows the scores obtained by Subject 4 on the DASS-21 subscales. The results show indicators of adequate mental health, although it is necessary to pay special attention to anxiety, which has high peaks at times.

## Discussion

The aim of this study was to determine the relationship between the subjective psychological disposition of the athlete who has just overcome a sports injury and the nature of the mood profile and mental health in order to predict the risk of recurrence.

### Subject 1 discussion

The obtained results showed that Subject 1 had the correct psychological disposition to resume sports. These results are consistent with other studies (Prapavessi, 2000; Neal et al., 2013; Clement et al., 2015; Caron et al., 2021) showing that athletes who adequately manage their emotions (Brewer et al., 2007; Glazer, 2009; García et al., 2015; Webster et al., 2018; Cools et al., 2021) are more successful in their athletic performance and return to RTP (De la Vega et al., 2013; Díaz et al., 2015; Horvath and Rothlin, 2018; Rollo et al., 2021). Similarly, the emotional profile of the iceberg described by Morgan (1980) has been gradually adopted, which is consistent with similar studies by other authors (Díaz et al., 2015; Andrade et al., 2016; Moreno-Tenas, 2018; Arroyo del Bosque et al., 2020; Palomo, 2020). Together with the low anxiety, stress and depression scores, this suggests that these are emotional scores associated with an effective mental health model to predict athletic success. Based on the existing literature indicating that a timely return to sports practise, a correct psychological predisposition to RTP, and appropriate emotional management prevent the possible ocurrence of recurrences, the data obtained were favorable for RTP and avoid future recurrences (Lu and Hsu, 2013; Neal et al., 2013; Roy et al., 2015; Gomez-Espejo et al., 2018; Moreno-Tenas, 2018; Green et al., 2020; Zhang et al., 2022).

### Subject 2 discussion

The results indicated that further testing was needed to determine if the athlete was psychologically prepared for the RTP. However, the mood profile was consistent with the iceberg profile, which states that mood did not change prior to the competition because it was a healthy psychological state that was maintained both after the competition and after practices. These results are consistent with other studies (De la Vega et al., 2011; Díaz et al., 2015; Andrade et al., 2016; Moreno-Tenas, 2018; Arroyo del Bosque et al., 2020; Palomo, 2020). On this basis, and with low levels of anxiety, depression, and stress, it seems to show indicators of good mental health. Different studies have shown that negative emotions tend to decrease as the return to sport process progresses, while positive emotions tend to increase as the RTP process progresses (Lalín, 2009; Mainwaring et al., 2010; Olmedilla et al., 2014; García et al., 2015; Rossi et al., 2021). Therefore, it is possible that she did not return to the sport with complete certainty, but that her perceptions changed as the competition evolved.

### Subject 3 discussion

The results of the PRIA indicated that the athlete felt psychologically unable to return to sport or that further complementary testing was needed to confirm this. In addition, the inverted iceberg profile he showed in Game 1, along with the increase in anxiety and stress scores over the course of the evaluation, indicated that the athlete was not ready to return to the sport. With this in mind, and considering that positive mood states serve as indicators of protection against sports injuries and recurrences (Rozen and Horne, 2007), the likelihood of this athlete relapsing is high.

### Subject 4 discussion

He demonstrated adequate psychological predisposition to return to sport safely and showed a good mood profile consistent with the iceberg profile. The mood profile for Match 1 and Training Week 1 did not show the iceberg profile as strongly. In the first case, high scores were shown in the Tension, Anger and Fatigue factors (without exceeding the central point), whereas in training, high scores were reported in the Fatigue factor. These results are consistent with those of Alzate et al. (2004), who demonstrated that athletes increasingly adopted an iceberg profile as the recovery process progressed (Abenza et al., 2009), highlighting that the emotional response to sports injury is not a static phenomenon and that the effectiveness of sports rehabilitation treatments can be enhanced by formal or informal assessments of changes in the athlete’s mood during the rehabilitation period (Arroyo del Bosque et al., 2020). Finally, the low scores of the depression factor decreased as the assessment progressed, while, the scores for anxiety and stress fluctuated. These results may be due to the fact that, as suggested by Walker et al. (2010), the term anxiety or stress in relation to a new injury would be more appropriate to refer to the emotional response traditionally known as fear of a new injury, because from a psychological perspective, the RTP phase can be particularly challenging as anxiety and stress may resurface once the athlete has been cleared for RTP (Clement et al., 2015; Green et al., 2020).

Previous literature has shown that returning athletes to sport before they are psychologically ready can lead to fear, anxiety, stress, recurrence, second injuries, depression, and decreased performance (Ahern and Lohr, 1997; Clement et al., 2013; Rossi et al., 2021).

## Future lines of research

This study has some limitations that should be considered. First, the sample is insufficient and geographically very limited, since it was conducted only in one autonomous community. The fact that a sample of soccer players from different areas (11-a-side soccer and futsal) was studied. While there are enough studies for 11-a-side soccer, this is not the case for futsal, so it could be very interesting to open a line in this field. In this sense, it seems reasonable to use samples as homogeneous as possible. Also, the exceptional situation imposed by COVID-19, which forced the interruption of competitions and training, forced to stop the evaluation process. In conclusion and considering these limitations, it would be necessary to consider in future researches the continuity of this work and try to expand the study population and its homogeneity. In addition, it would be interesting to monitor the athlete during his RTP process to ensure an adequate return to sports practice and to check if recurrences occur.

At an applied level, the results presented provide new information for the design of intervention programs aimed at coaches (Sousa et al., 2007; Soriano et al., 2014) and psychologists (De la Vega et al., 2011; Díaz et al., 2015; Moreno-Tenas, 2018; Arroyo del Bosque et al., 2020; Caron et al., 2021). Proper reading of moods as well as anxiety, stress, depression, and psychological predisposition can help sport professionals determine the right time for RTP. In this way, this work calls for the attention of technicians, coaches and clubs to integrate the psychological variables in their training programs, just as they train the physical, conditioning, technical and tactical aspects and take measures that help the injured athlete to develop a realistic and positive attitude toward rehabilitation as a guarantee for a successful recovery.

## Data availability statement

The datasets presented in this study can be found in online repositories. The names of the repository/repositories and accession number(s) can be found in the article/Supplementary material.

## Ethics statement

The studies involving human participants were reviewed and approved by Ethics Committee of the University of Murcia. The patients/participants provided their written informed consent to participate in this study.

## Author contributions

VG-E, AO, and EO designed the study as a whole. AO designed the questionnaires’ protocol and VG-E adapted them in the online version. VG-E prepared the draft of the introduction, with all the coauthors contributing to the revision and final version. VG-E carried out the data collection. EO was in charge of the statistical analyzes. LA-C and AG-M prepared the first draft of the discussion, with all the co-autothors contributing to the final version and revisions. All authors contributed to the article and approved the submitted version.
