# Supplementary material for: Psychological readiness to return to sports practice and risk of recurrence: Case studies
Source: Front Psychol. 2022 Sep 23;13:905816. doi: 10.3389/fpsyg.2022.905816 (PMC9540195; doi:10.3389/fpsyg.2022.905816)
Supplement: Supplementary file 1 [file Data_Sheet_1.PDF]

Anex I: Questionnaire of personal and sports variables

<table>
<tr><td>Name and surname</td><td colspan="7"></td></tr>
<tr><td colspan="2">Phone number</td><td colspan="2"></td><td>E-mail</td><td colspan="3"></td></tr>
<tr><td>Age</td><td></td><td>Sex</td><td></td><td>Sport</td><td colspan="3"></td></tr>
<tr><td>Date</td><td></td><td>Sport club</td><td colspan="5"></td></tr>
<tr><td>Game position</td><td colspan="7"></td></tr>
<tr><td colspan="3">Sports category</td><td colspan="5"></td></tr>
<tr><td colspan="2">Nº of years in the category</td><td></td><td colspan="4">Nº of years practicing sports regularly</td><td></td></tr>
<tr><td colspan="3">Training days per week</td><td></td><td colspan="3">Training time per day</td><td></td></tr>
</table>

Anex II: Sports injury history

INSTRUCTIONS: Te pedimos que completes este cuestionario sobre las lesiones padecidas en las 2 últimas temporadas**.**

1. **¿Cuántas lesiones padeciste la temporada pasada? _____**
2. **Indica para cada lesión el tipo y la gravedad de la misma**

Lesión 1:

TIPO	MUSCULAR	FRACTURA	TENDINITIS	CONTUSIÓN	ESGUINCE	OTRAS

GRAVEDAD (Marcar la que corresponda)			
**LEVE**<br>Requiere tratamiento y, al menos, 1 día sin entrenamiento	**MODERADA**<br>Requiere tratamiento 6 días o más sin entrenamiento y pérdida de algún partido	**GRAVE**<br>Requiere de uno a tres meses de baja deportiva; a veces de hospitalización e incluso intervención quirúrgica	**MUY GRAVE**<br>Requiere más de 4 meses de baja deportiva; a veces, produce disminución permanente del rendimiento deportivo

Lesión 2:

TIPO	MUSCULAR	FRACTURA	TENDINITIS	CONTUSIÓN	ESGUINCE	OTRAS

GRAVEDAD (Marcar la que corresponda)			
LEVE	MODERADA	GRAVE	MUY GRAVE

Lesión 3:

TIPO	MUSCULAR	FRACTURA	TENDINITIS	CONTUSIÓN	ESGUINCE	OTRAS

GRAVEDAD (Marcar la que corresponda)			
LEVE	MODERADA	GRAVE	MUY GRAVE

Lesión 4:

TIPO	MUSCULAR	FRACTURA	TENDINITIS	CONTUSIÓN	ESGUINCE	OTRAS

GRAVEDAD (Marcar la que corresponda)			
LEVE	MODERADA	GRAVE	MUY GRAVE
